# Secreted Protein Acidic and Rich in Cysteine Mediates the Development and Progression of Diabetic Retinopathy

**DOI:** 10.3389/fendo.2022.869519

**Published:** 2022-06-03

**Authors:** Liying Luo, Xi Sun, Min Tang, Jiahui Wu, Tianwei Qian, Shimei Chen, Zhiyuan Guan, Yanyun Jiang, Yang Fu, Zhi Zheng

**Affiliations:** ^1^ Department of Ophthalmology, Tongren Hospital, Shanghai Jiao Tong University School of Medicine, Shanghai, China; ^2^ Department of Hematology, Shanghai General Hospital, Shanghai Jiao Tong University School of Medicine, Shanghai, China; ^3^ Department of Ophthalmology, Shanghai General Hospital, Shanghai Jiao Tong University School of Medicine, Shanghai, China; ^4^ Shanghai Key Laboratory of Ocular Fundus Diseases, Shanghai Engineering Center for Visual Science and Photomedicine, Shanghai, China; ^5^ Department of Orthopedics, The Shanghai Tenth People’s Hospital of Tongji University, Shanghai, China

**Keywords:** SPARC, diabetic retinopathy, integrin β1, angiogenesis, HRCECs

## Abstract

**Backgrounds:**

Diabetic retinopathy (DR) is one of the most severe microvascular complications of diabetes mellitus (DM). Secreted protein acidic and rich in cysteine (SPARC) has been found to play an important role in many diseases, but its role and mechanism in DR remain unknown.

**Methods:**

We studied the role of SPARC and integrin β1 in vascular pathophysiology and identified potential therapeutic translation. The SPARC levels were tested in human serum and vitreous by ELISA assay, and then the Gene Expression Omnibus (GEO) dataset was used to understand the key role of the target gene in DR. In human retinal capillary endothelial cells (HRCECs), we analyzed the mRNA and protein level by RT-PCR, immunohistochemistry, and Western blotting. The cell apoptosis, cell viability, and angiogenesis were analyzed by flow cytometry, CCK-8, and tube formation.

**Results:**

In this study, we investigated the role of SPARC in the development and progression of human DR and high glucose-induced HRCEC cells and found that the SPARC-ITGB1 signaling pathway mimics early molecular and advanced neurovascular pathophysiology complications of DR. The result revealed that DR patients have a high-level SPARC expression in serum and vitreous. Knockdown of SPARC could decrease the expressions of inflammatory factors and VEGFR, inhibit cell apoptosis and angiogenesis, and increase cell viability by regulating integrin β1 in HRCECs.

**Conclusion:**

SPARC promotes diabetic retinopathy *via* the regulation of integrin β1. The results of this study can provide a potential therapeutic application for the treatment of DR.

## Introduction

Diabetic retinopathy (DR) is one of the most common microvascular complications of diabetes (types 1 and 2) and remains the leading cause of blindness and vision impairment worldwide (1). The global prevalence of DR is expected to remain high through 2034 in DR screening, treatment, and public healthcare strategies (2). In addition, DR is more common in women than men with type 2 diabetes, but men have more severe retinopathy, blurred vision, or blindness (3). The process consists of multiple events. In the early stages of diabetic retinopathy, hyperglycemia and altered metabolic pathways lead to oxidative stress and neurodegeneration (4). Ophthalmoscopy revealed a rupture of the blood–brain barrier, the release of various inflammatory cytokines and plasma proteins, and solid exudates (5). Therefore, when studying pathogenesis, more attention should be paid to molecular biology and molecular interventions targeting the disease. Understanding the changes and molecular biological signaling pathways of DR may represent valuable therapeutic targets for DR treatment.

Secreted protein acidic and rich in cysteine (SPARC, BM-40, osteonectin) is a stromal cellular protein (binding to the extracellular matrix) widely expressed in ocular tissue with multiple roles, including metabolic homeostasis, inflammation reduction, extracellular matrix remodeling, and collagen maturation (6). SPARC modulates tissue physiology by altering cell-ECM interactions, cell proliferation, and migration. Due to these properties, SPARC is involved in wound healing, angiogenesis, tumorigenesis, and inflammation (7). SPARC promotes these functions by affecting the activity of cytokines and growth factors such as vascular endothelial growth factor (VEGF), the most potent and widespread angiogenic mitogen in capillary endothelial cells (8). SPARC KO mice presented impaired glucose homeostasis and insulin section capacity and SPARC was confirmed as a key factor in the pathogenesis of diabetes (9). SPARC plays an important role in hyalocyte-to-myofibroblast transdifferentiation in proliferative diabetic retinopathy, and deletion of SPARC enhances retinal vaso-obliteration (10, 11). However, research has shown that the role of SPARC in the function of DR in humans and cellular is still lacking, and the exact mechanistic link between SPARC and disease development in DR has not been fully explored.

SPARC has been shown to modulate the mitogenic activity in normal endothelial cells in a dose-dependent manner (12) and play dual roles in tumor angiogenesis and tumor extravasation and mediate permeability that is related to endothelial barrier function (13). The expression of SPARC was significantly correlated with the expression of VEGF in colon tumors (14). SPARC regulates glioma growth by altering the tumor microenvironment and inhibiting tumor angiogenesis by suppression of VEGF expression and secretion (15). Furthermore, SPARC-secreted glycoprotein prevents deleterious cardiac inflammation by improving glycocalyx and endothelial barrier functions during viral myocarditis (16). However, the relationship between SPARC and angiogenesis and inflammation has not been investigated in DR.

Integrin β1 (ITGB1) belongs to the β-subfamily and forms dimers with multiple α-subunits. Previous studies found that inhibition of ITGB1 and focal adhesion kinase (FAK) inhibits the migration of bovine myeloid-derived suppressor cells (MDSCs) (17). In addition, ITGB1 signaling plays a significant role in pericyte apoptosis in DR (18). The high levels of glucose in diabetes increased VEGF expression in vascular endothelial cells through fibronectin and integrin β1 interaction (19). SPARC can affect the migration and differentiation of bovine muscle-derived satellite cells *via* the ITGB1-mediated cell signaling pathway (20). In the human lens epithelium-derived cell line SRA01/04, the expression of SPARC and ITGB1 exhibits as a molecular biology of lens cells (21).

This paper attempts to show the effect of SPARC expression in human samples and human cell lines. Our results suggest a unique mechanism for how SPARC-ITGB1 and hyperglycemia modulate VEGF signaling and inhibit neovascularization (NV) in DR. Thus, we aimed to provide direct and informative evidence of SPARC-ITGB1 expression and function involved in the development of DR.

## Materials and Methods

This study was approved by the ethics committee of Shanghai General Hospital and the institutional review board of the University (No. 2021-047). All patients were informed and signed an informed consent form. According to the CONSORT guidelines, these studies also comply with the Declaration of Helsinki.

### Patients

We performed a prospective nonrandomized study at the Department of Ophthalmology, Shanghai General Hospital, Shanghai Jiao Tong University School of Medicine from January to December 2021. DR patients (12 patients) included in this study were diagnosed with diabetic retinopathy requiring surgery, with or without vitreous hemorrhage. Among the 7 control patients, five had macular epiretinal membranes and two had a macular hole, and they need surgery at the same time without hypertension and diabetes mellitus (DM).

At our clinic, patients who were clinically assessed as eligible were asked if they were interested in participating in the study. Before applying, they must decide on whether they would like to participate or not, and if so, they should provide written informed consent.

### Inclusion and Exclusion Criteria

The minimum age of participants was 18 years old. Inclusion criteria included the diagnosis of diabetic retinopathy and either diabetes mellitus type 1 or type 2. The control group included patients diagnosed with macular epiretinal membranes or macular holes who needed pars plana vitrectomy.

Exclusion criteria included a history of subtotal or complete vitrectomy (3 port pars plana vitrectomy), treatment with an antiangiogenic agent, partial vitrectomy with drug administration, or laser coagulation within the 90 days before screening or treatment with a long-acting corticosteroid within the last 90 days before screening. In addition, patients who had undergone a cataract operation or posterior capsule opacification treatment within the past 90 days or had any other eye disease or clinically significant glaucoma evident at the time of screening were excluded. Patients with uncontrolled intraocular pressure (≥30 mmHg) were excluded.

Additional exclusion criteria included patients with uncontrolled hypertension (>160/90 mmHg (systole/diastole), poorly controlled diabetes (HbA1c >10%), or those diagnosed with an autoimmune disease. Patients were excluded if pregnant or breastfeeding during the study, if they had participated in a clinical trial within 30 days before screening, and if they had any other condition, that at the discretion of the investigator, was deemed to be inconsistent with participation in the trial. Finally, patients who reported drug or alcohol abuse within the 180 days before screening were excluded.

### Serum and Vitreous Sampling

A blood sample (5–10 ml blood) was taken before the surgery to determine the SPARC level. A minimally invasive 3-port partial pars plana vitrectomy was performed in all cases by one vitreoretinal surgeon in the operating room. The extracted vitreous sample was placed directly in the freezer at a temperature of −70°C. Once completed, the removal of the probe leaves a self-sealing wound, which reduces the risk of leakage from the eye and limits the penetration of pathogens from the outer ocular surface.

### Analysis of Serum and Vitreous Samples

Serum and vitreous samples were sent for laboratory analysis following surgery. The laboratory received no information about whether each sample was part of the DR or control group. This was to allow for laboratory analysis to be conducted in a blinded fashion. The SPARC level was detected using an enzyme-linked immunosorbent assay (ELISA) (DY941-05, R&D, Minneapolis, USA).

### HE Staining and Immunohistochemical Analysis

The sample of human proliferative membranes and epiretinal membranes were fixed in 2% PFA for 2 h and then frozen in isopentane (−55°C). Next, the eyes were embedded in paraffin and cut into 5-μm-thick sections, which were then mounted on glass slides. Paraffin-embedded sections were dewaxed with xylene, washed by gradient alcohol or distilled water, and stained with hematoxylin for 1–3 min. Sections were then differentiated by 1% hydrochloric alcohol, turned blue by PBS, stained by eosin, and dehydrated using gradient alcohol. After permeabilization with xylene, the tissues were photographed under microscopy (BX53, Olympus, Japan).

In addition, the tissues were processed for immunohistochemistry (IHC) analysis using an anti-SPARC antibody (ab225716, Abcam, Cambridge, the UK). Before the IHC procedure, sections were deparaffinized and rehydrated by immersing the slides in xylene and alcohol at descending concentrations. Colocalization of individual SPARC, TRIB3, and BRN3A, or GFAP and vimentin proteins in the retinal sections were detected using fluorescent confocal microscopy. Fluorescence intensity was measured using ImageJ software. Absorption control was performed using the recombinant human SPARC protein.

### GEO Dataset and Data Processing

The RNA-sequencing data of DR patients and corresponding clinical information were obtained from the GEO dataset (GSE102485) on December 31, 2021. mRNA expression data of 11 DR patients and 3 healthy people were collected.

### Difference Gene Expression and Functional Enrichment Analysis

The differential expression of mRNA in DR tissue and normal control samples was evaluated using the R software (Version 3.8; http://www.bioconductor.org/packages/release/bioc/html). The heatmap and volcano of these genes were plotted using the R software. Pearson correlation analyses were performed to identify gene-to-gene correlation. The *p*-value of 0.05 was considered the significant threshold in all tests.

### GO and KEGG Analyses

Gene ontology (GO), including the biological process (BP), cellular component (CC), and molecular function (MF) categories, was conducted with the “ggplot2” package in the R software. Similarly, this package was also utilized to perform the Kyoto Encyclopedia of Genes and Genomes (KEGG) analysis.

The STRING website (https://string-db.org/) uses the protein–protein interaction network to study the interaction between protein names (SPARC) and organisms (*Homo sapiens*). The most important parameters are defined as follows: the minimum required interaction rating [low confidence (0,150)], the effectiveness of the network edge, the maximum number of interactions to be displayed (no more than 50 first shells), and the active interaction sources.

To further the function of SPARC, we used GeneMANIA tools (http://genemania.org/) to understand the coexpression, colocation, genetic interaction, pathway, physical interaction, and predicted and shared protein domains of SPARC.

### Establishment of High Glucose-Induced Cell Model

Human retinal capillary endothelial cells (HRCECs; PriCells Biotechnological Co. Ltd., Wuhan, China) were cultured in DMEM (Gibco, Grand Island, NY, USA), 10% fetal bovine serum (FBS, Gibco), 100 IU/ml penicillin, and 100 μg/ml streptomycin (Gibco). All cells were incubated in a humidified atmosphere at 37°C under 5% CO_2_ air. Subculturing was performed every 2 days, and cells were seeded into 96-well plates with the density of 4 × 105 cells/ml, cultured for 1 h, and detached with 0.25% trypsin. d-Glucose was added to the medium at a final concentration of 30 mmol/L to generate high-glucose (HG) conditions, while cells treated with DMEM (low glucose) with 5 mmol/L served as the low-glucose group. In addition, cells cultured in basal DMEM supplemented with 25 mmol/L glucose served as the normal medium conditions (control group).

### siRNA and Plasmid Transfection in HRCECs

Overexpression plasmids for SPARC and ITGB1 as well as the silencing plasmids sh-NC, sh-SPARC, and sh-ITGB1 were synthesized by Shanghai GenePharma Biological Co. Ltd. (Shanghai, China). CDS sequences of SPARC and ITGB1 were obtained from NCBI, followed by polymerase chain reaction (PCR) amplification. Plasmids were established by cloning the corresponding sequences of SPARC to pcDNA3.1 (+) vector through restriction enzyme sites. Confluent cells were then transfected with 1 μg sh-SPARC plasmid according to the manufacturer’s protocol in the Lipofectamine 2000 kit (Invitrogen, Carlsbad, CA, USA). Meanwhile, cells received treatment with vector + dimethyl sulfoxide (DMSO), sh-SPARC + DMSO, sh-ITGB1 + DMSO, and sh-SPARC + sh-ITGB1, followed by treatment with DMSO and 0.5 or 1.0 μM transmethylase inhibitor 5-Aza (Sigma) solution.

HRCECs were cultured at a density of 4 × 105 cells per 6-cm well, and they were 80%–90% confluent at the time of transfection. Two micrograms of each plasmid was transfected with 4 μl lipofectamine 2000 reagent (Invitrogen, Karlsruhe, Germany) according to the manufacturer’s recommendations, and the empty vector was transfected into HRCECs as a control for comparison.

### Cell Viability Assay

A Cell-Counting Kit-8 (CCK-8) assay was used to evaluate the cell viability. HRCECs were treated with sh-SPARC for 24 h. The cells were plated into 96-well plates for 48 h in the incubator. At the end of the incubation period, 10 μl CCK-8 solution was added to each well, and the plates were returned to the incubator for an additional 2 h at 37°C. Absorbance was measured at 450 nm using a microplate reader.

### Tube Formation Assay

Tube formation assay was performed as we previously described (22). HRCECs were placed on the Matrigel and treated with sh-SPARC for 12 h. Tube formation was quantified by counting the number of connected cells in randomly selected fields and dividing by the total number of cells in the same field.

### Western Blotting and RT-PCR Analyses

HRCECs were harvested, and total proteins were obtained using RIPA Lysis Buffer (Beyotime, Shanghai, China). The protein concentration was determined using the BCA Protein Quantitation kit (Beyotime, Shanghai, China). The proteins were then separated by SDS-PAGE and transferred to PVDF membranes. The membranes were blocked with 5% nonfat milk and 0.1% Tween-20 for 1 h at room temperature. Samples were then incubated with the primary antibodies overnight at 4°C, which were the primary antibodies against SPARC (66426-1-Ig, Proteintech), ITGB1 (ab199056 Abcam), vascular endothelial growth factor receptor (VEGFR) (ab134191, Abcam), integrin-linked kinase (ILK) (ab134179, Abcam), and fibronectin 1 (FN1) (ab52480, Abcam). After being washed three times with TBST buffer, the membranes were incubated with secondary antibodies (ab150077, Abcam) at 4°C for 3 h. Enhanced chemiluminescence (ECL) reaction reagents were used for visualization, and ImageJ software was employed to analyze the results. The protein bands were quantified and normalized to the expression of glyceraldehyde 3-phosphate dehydrogenase (GAPDH).

The total RNA was extracted using Trizol reagent (TaKaRa, Dalian, Liaoning, China), and the cDNA was synthesized with RNA Transcription Kit (TaKaRa). RT-qPCR was then performed using the SYBR Green RT-qPCR system. SPARC, ITGB1, VEGFR, ILK, and FN1 mRNA expression were tested in our study mRNA expression were tested in our study. The relative expressions of mRNAs were evaluated by the 2−ΔΔCq method, and the internal normalization control is GAPDH (**Supplementary Table S1**).

### Flow Cytometry Apoptosis Detection Assay

HRCECs were seeded in 6-well plates, then collected by trypsinization, washed in 4°C PBS, and trypsinized into cell suspensions. Phosphatidyl-serine (PS) exposure due to the flipping of the plasma membrane, a concomitant feature during apoptosis, was evaluated by PE Annexin V Apoptosis Detection Kit (559763, BD Pharmingen) by flow cytometry analysis containing PE Annexin V and 7-AAD. The experiments were independently repeated three times.

#### Statistical Analysis

All results are expressed as the mean ± standard deviation (SD). Statistical differences were assessed by a 2-tailed Student’s *t*-test or one-way ANOVA statistical analysis followed by Tukey’s test. All results were repeated at least three times, and *p* < 0.05 was considered significant.

## Results

### SPARC Is Highly Expressed in DR Patients’ Serum, Vitreous, and Proliferative Membranes

**Figure 1:**
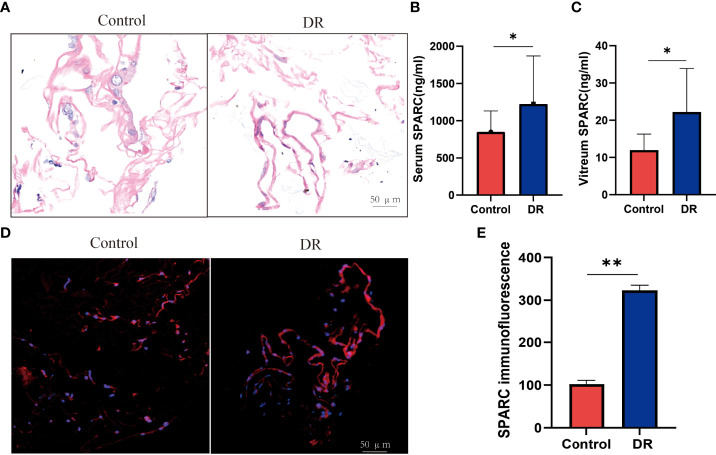
The SPARC level is increased in diabetic retinopathy patient. **(A)** HE staining. **(B, C)** Serum and vitreum SPARC level. **(D, C)** Immunofluorescence staining of SPARC in control and diabetic retinopathy patient. **(E)** SPARC immunofluorescence between Control and DR group. *p < 0.05,**p < 0.01.

Recent studies found that dysregulation of SPARC is associated with a variety of obesity-related diseases, including type 2 diabetes and its complications related to obesity, kidney and liver disease, cardiovascular disease, and cancer (23). Therefore, we investigated the SPARC expression in human serum and vitreous samples and histological results in proliferative membranes. The demographic data of the participants are shown in **Table 1**. HE staining showed that the epiretinal membrane is mainly composed of extracellular matrix (ECM) such as collagen fiber, and the nucleus is round and spindle shaped in the control group. DR group proliferation membrane is mainly manifested as vascular fiber membrane, which is characterized by more luminal structures surrounded by endothelioid cells (**Figure 1A**). To this end, we analyzed and quantitated SPARC level and immunoreactivity. Overall, the serum and vitreum SPARC levels in the DR group were highly increased significantly than in the control group (**Figures 1B, C** **)**. Representative control and DR of a 67-year-old and 56-year-old man are shown in **Figure 1D**. Strong evidence of DR immune responses in retinal endothelial cells, ganglia, and photoreceptor cells was observed in a 67-year-old nondiabetic man compared with the control (**Figure 1E**).

**Table 1:** Demographic and clinical characteristics of patients.

Characteristics	Total	Control^a^	DR	*p*-value
Patients (No.)	19	7	12	–
Age (year)	57.8 ± 10.0	64.9 ± 6.5	52.8 ± 9.2	0.0940
Man/woman (%)	37 (7/19)	29 (2/7)	42 (5/12)	0.5681
Positive history of hypertension (%)	11 (2/19)	0 (0/7)	12 (2/17)	0.3432
Positive history of diabetes (%)	63 (12/19)	0 (0/7)	100 (12/12)	<0.0001^*^
SPARC (ng/ml)	17.12 ± 9.28	11.98 ± 4.31	22.22 ± 11.65	0.0405^*^

a Diagnosed as macular hole or epiretinal membrane. DR, diabetic retinopathy; SPARC, secreted protein acidic and rich in cysteine. ^*^Significant results.

### Differential Gene Expression and Pathway Signaling of SPARC

**Figure 2:**
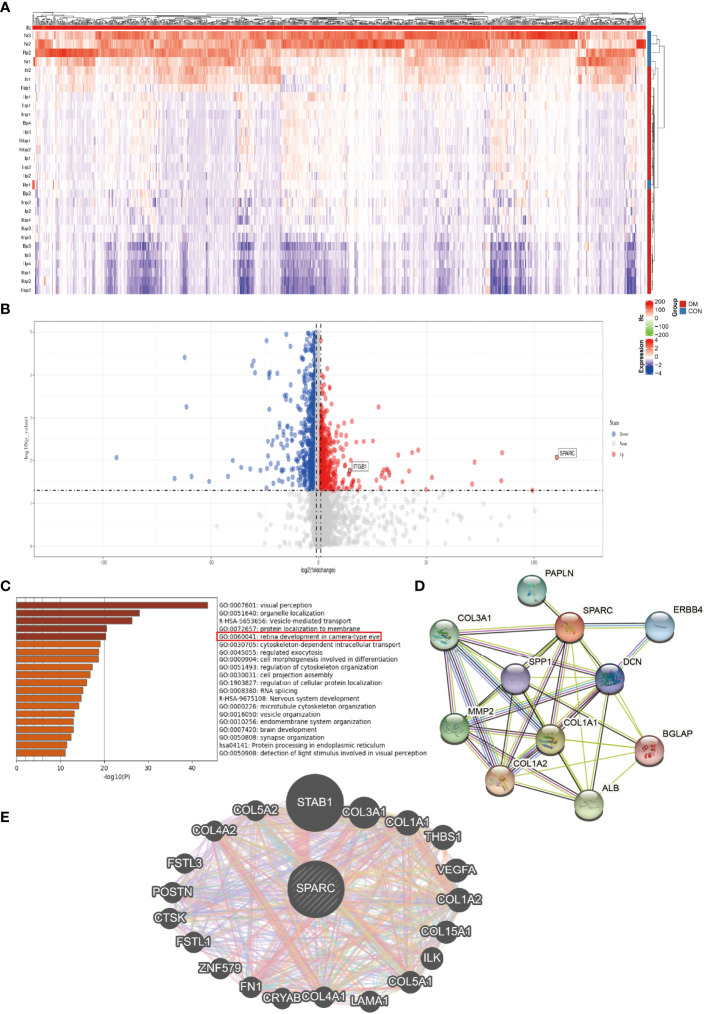
The bioinformation analysis by GEO dataset. **(A)** Heatmap of DR patient. **(B)** Volcano map of DR patient. **(C)** GO pathway signaling. **(D)** Protein–protein network by the STRING analysis. **(E)** Target gene network by the GeneMANIA tool.

Further analysis showed the differential gene expression of DR patients in the GEO dataset. The heatmap and volcano map are shown in **Figures 2A** **, B**. SPARC and ITGB1 expressions are significant differences between DR patients and healthy patients. GO disease analysis also found that SPARC was correlated with electroretinogram abnormality, photophobia, rod-cone dystrophy, and disorder of the eye (**Supplementary Figure S1B**). GO target gene analysis investigated ZNF513, AUTS2, ZBTB44, and SUPT16H (**Supplementary Figure S1C**), and GO-regulated gene analysis also found that NRL, ETV7, PAX6, and CRX can regulate the expression of SPARC (**Supplementary Figure S1C**). GO enrichment and GO network found that SPARC plays an important role in retinal development in the camera-type eye (**Figure 2C**; **Supplementary Figure S1A**). The GeneMANIA network showed that SPARC significantly correlated with STAB1, FN1, VEGF, and ILK (**Figure 2C**). Protein–protein network also investigated the correlation of SPARC with ERBB4, ALB, COL1A1, and MMP2 (**Figures 2D, E**).

**Figure 3:**
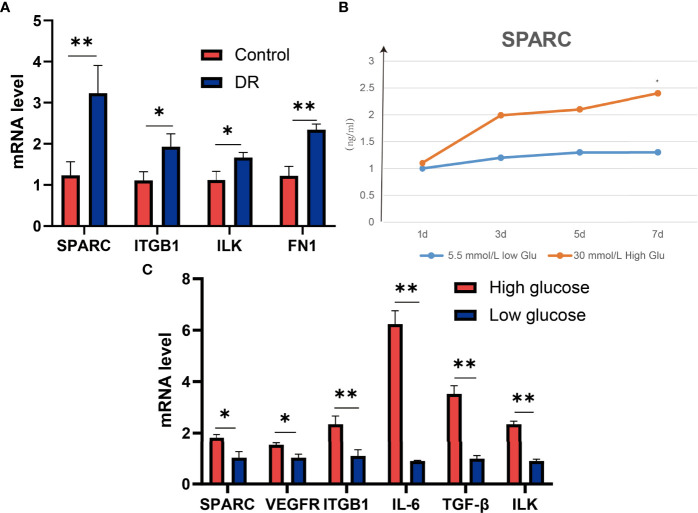
SPARC regulates the expression of ITGB1, inflammation, and VEGF genes in high-glucose-induced HECEC cells. **(A)** The mRNA expression of SPARC, ITGB1, ILK, and FN1 in DR and control tissues. **(B)** SPARC level in different glucose-induced HECEC cells. **(C)** The mRNA expression in different glucose-induced HECEC cells. *p < 0.05,**p < 0.01.

To verify these results, we test the mRNA of SPARC, ITGB1, ILK, and FN1 levels in DR and control tissues and found that the DR group has higher SPARC, ITGB1, ILK, and FN1 mRNA levels than the control group (**Figure 3A**). It is indicated that a high SPARC level is correlated with the development of DR, and the SPARC expression level also links to ITGB1 and ILK.

### SPARC Is Highly Expressed in HG-Induced Cell Models

HG-induced retinal pigment epithelium can stimulate the development of DR. Therefore, we also analyzed the SPARC level in a different dose of HG-induced cell models. We found that the SPARC level can be increased significantly in HG medium (30 mmol/L) in than low glucose medium (5.5 mmol/L) after 7 days of incubation, and the effect of HG treatment on SPARC showed a dose-dependent manner. qPCR assay showed that the HG environment can also increase the SPARC, VEGFR, ITGB1, IL-6, TGF-β, and ILK mRNA levels than the low-glucose group (**Figure 3**). In this part, we further demonstrated that the expression of SPARC and related genes and proteins in the HRCEC cells were significantly increased under HG condition.

**Figure 4:**
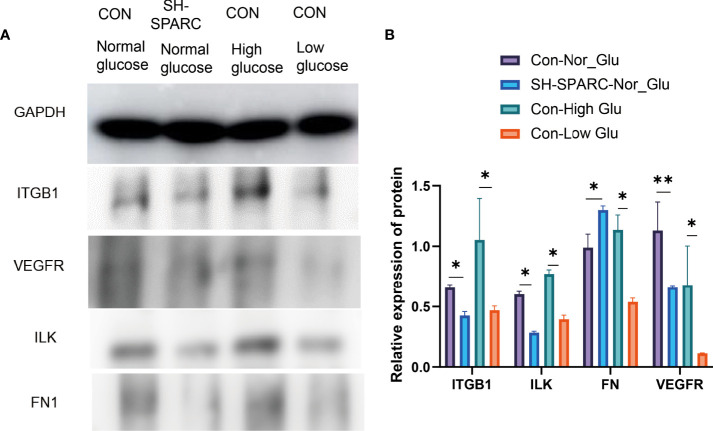
The protein levels in HRCEC cells under high glucose and low glucose, sh-SPARC cells in normal condition, and control group. **(A)** Representative figure. **(B)** The expression of proteins such as ITGB1, ILK, FN1, and VEGFR in HECEC cells. *p < 0.05,**p < 0.01.

In addition, we also analyzed the protein level in the HG-induced HRCEC cell model. The HG environment increased the expression of ITGB1, ILK, FN1, and VEGFR compared with the low-glucose-induced HRCEC cells (**Figure 4A, B** **)**.

### Ablation of SPARC Suppresses ITGB1/ILK mRNA and Endothelial and Inflammation Cell Biomarkers

**Figure 5:**
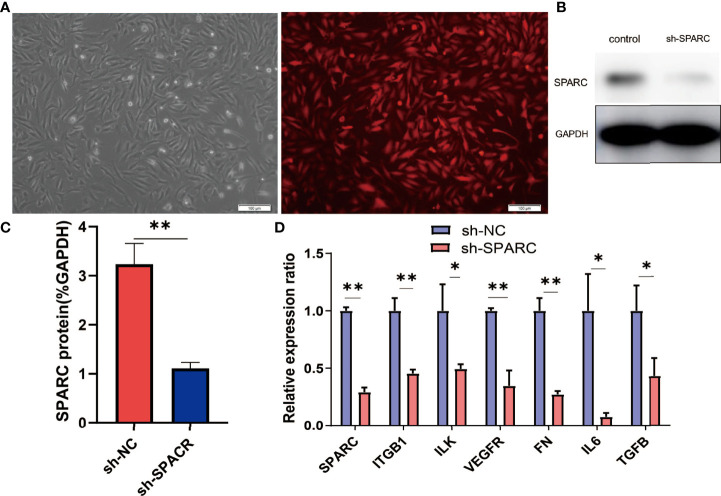
The related gene expression in SPARC gene silencing experiments. **(A)** Representative fluorescence figure. **(B**, **C)** Protein level of SPARC in SPARC gene silencing experiments. **(D)** The mRNA level in SPARC gene silencing experiments. *:*p* < 0.05,**:*p* < 0.01.

Subsequently, the study aimed to understand the role of SPARC and SPARC-related genes such as ITGB1 in the development of DR. We first transfected HRCECs with the sh-SPARC-interfered lentiviruses carrying red fluorescent protein. A representative figure of immunofluorescence has been shown in **Figure 5A**. The SPARC protein level has also been tested by Western blotting and was found to be significantly lower in the sh-SPARC group than in the control group, showing that the SPARC silence model is successful (**Figure 5**).

After the establishment of the cell silencing model, we detected the changes in SPARC-related gene expression. Firstly, the SPARC mRNA level was significantly decreased in the sh-SPARC group than in the sh-NC group, and then, the ITGB1, ILK, VEGFR, FN1, IL6, and TGF-β mRNA levels also markedly decreased in the sh-SPARC than in the sh-NC group.

Consisting with the mRNA expression of SPARC-related genes, SPARC knockdown could decrease the protein level of ITGB1, ILK, FN1, and VEGFR significantly than the control group (**Figure 4A, B** **)**.

### Overexpression and Ablation of ITGB1 in HG-Induced HRCEC Cells Suppress ITGB1/ILK mRNA and Endothelial and Inflammation Cell Biomarkers

ITGB1 overexpression and ablation demonstrate that the former can significantly increase the expression of VEGFR, FN1, IL6, and TGF-β in mRNA levels, whereas the latter could decrease the expression of VEGFR, FN1, IL6, and TGF-β in mRNA levels.

**Figure 6:**
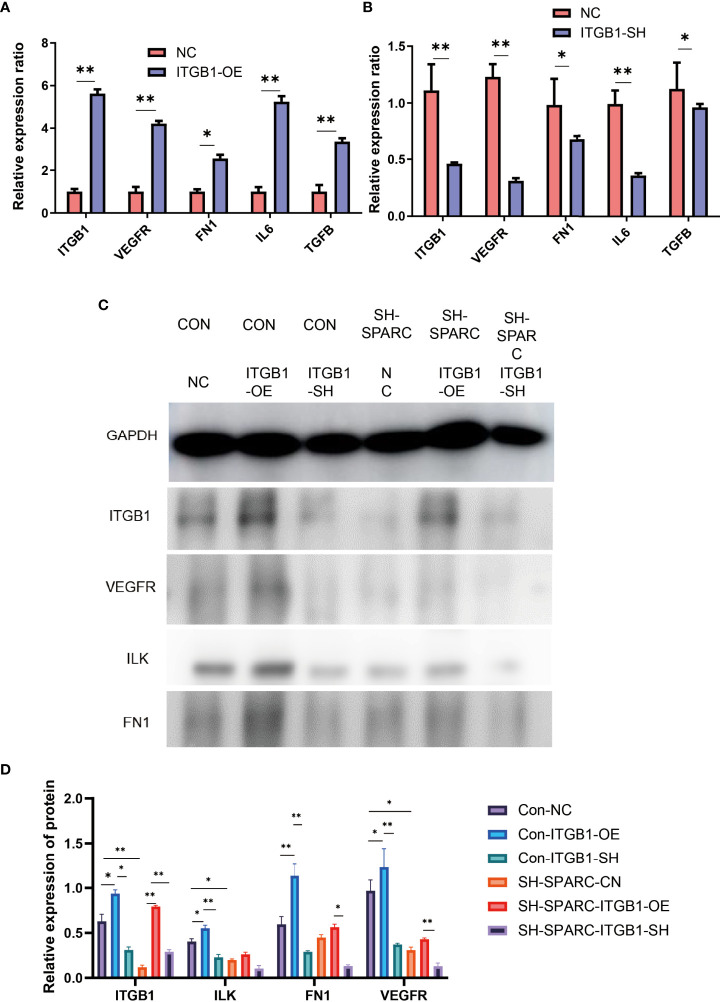
The mRNA and protein levels in overexpression and ablation of ITGB1 experiments. **(A)** Overexpression of ITGB1 experiments. **(B)** Ablation of ITGB1 experiments. **(C**, **D)** Protein level in the overexpression and ablation of the ITGB1 experiments. *:*p* < 0.05,**:*p* < 0.01.

In addition, the ITGB1 overexpression or ablation could increase or significantly decrease the protein expression levels of ILK, FN1, and VEGFR. The sh-SPARC/ITGB1-OE group could also increase the ITGB1 protein level while the sh-SPARC/ITGB1-sh group decreased the ITGB1 protein level significantly. However, sh-SPARC/ITGB1-OE could only increase the protein level of FN1 and VEGFR compared with the sh-SPARC/ITGB1-sh group (**Figure 6**).

### Ablation of SPARC and ITGB1 Inhibits the HG-Induced Apoptosis, Cell Viability, and Tube Formation of HRCECs

**Figure 7:**
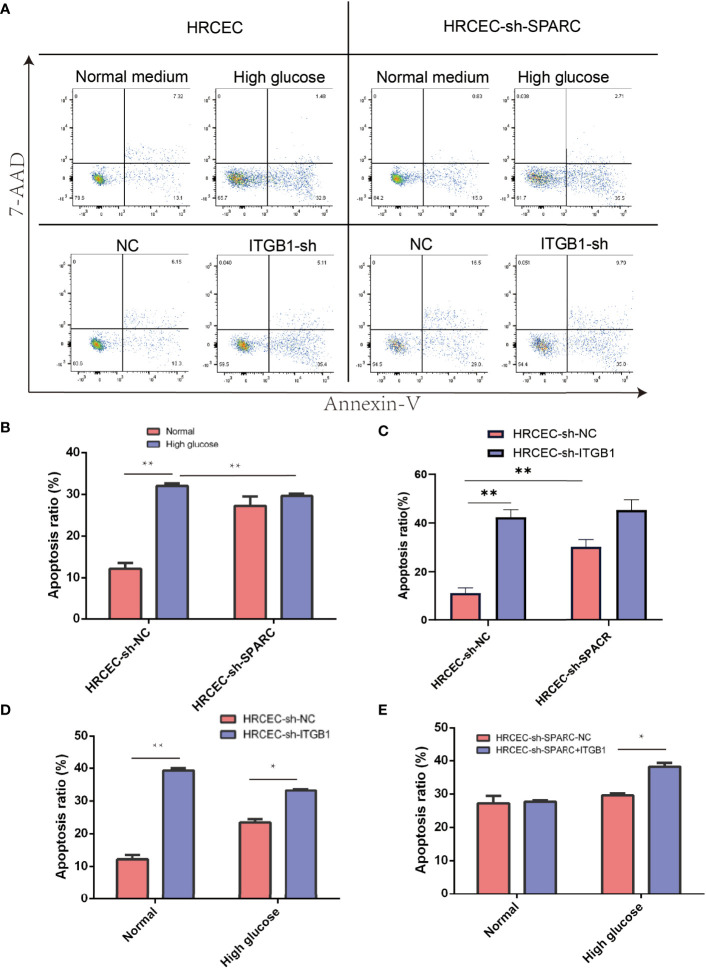
Apoptosis level in ablation of SPARC and ITBG1 in different glucose-induced experiments. **(A)** Representative figure of flow cytometry. Annexin V/7-AAD assay was performed to determine the percentage of apoptotic cells. **(B–E)** Apoptosis ratio in ablation of SPARC and ITBG1 in different glucose-induced experiments. *:*p* < 0.05, **:*p* < 0.01.

The cell apoptosis assay was conducted, and HG-induced HRCECs had a high apoptosis ratio when compared with the control group (**Figure 7A**). Firstly, HG-induced HRCECs can increase apoptosis levels in the control group more than normal glucose, and sh-SPACR can significantly decrease the apoptosis ratio in HG levels more than normal glucose (**Figure 7B**). In normal and HG (**Figure 7D**), the sh-ITGB1 group increased the apoptosis ratio significantly more than the sh-NC. In addition, sh-SPARC-ITGB1 also increased apoptosis significantly more than in the sh-SPARC-NC in HG (**Figure 7E**).

**Figure 8:**
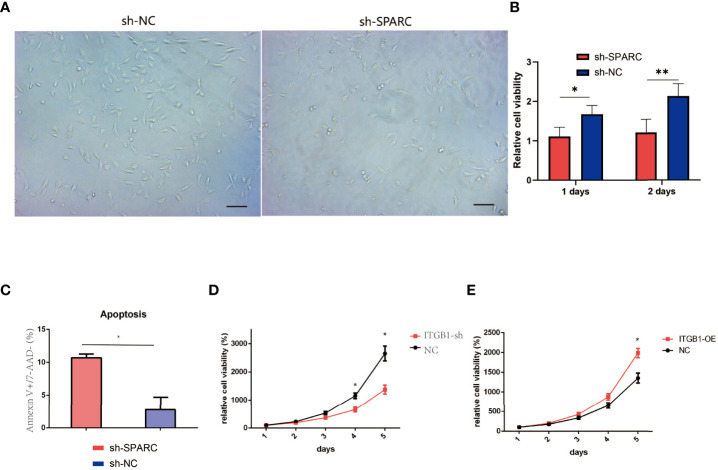
Cell viability experiments test by CCK-8 analysis. **(A)** Representative figure of cell viability experiment. **(B)** The cell viability experiments in ablation of SPARC in 1 and 2 days. **(C)** Apoptosis of Annexin V+/7-AAD− experiment. **(D**, **E)** The cell viability experiments in 1, 2, 3, 4, 5 days with overexpression and ablation of ITGB1. *:*p* < 0.05, **:*p* < 0.01.

**Figure 9:**
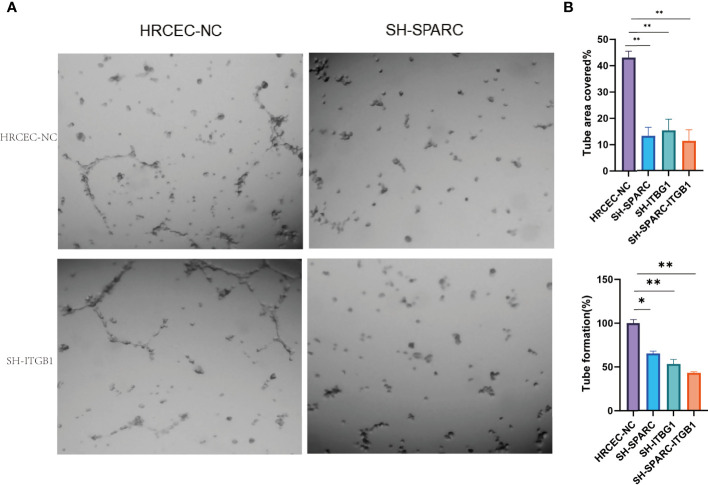
The tube formation of ablation of SPARC and ITGB1 experiments. **(A)** Representative figure of tube formation. **(B)** Tube formation area covered. **(C)** Tube formation rate. *:*p* < 0.05,**:*p* < 0.01.

We analyze the cell viability by CCK-8 tests in **Figure 7A**. After 1 or 2 days postincubation (**Figures 8A, B**), sh-SPARC significantly decreased cell viability more than the sh-NC group (**Figures 8C–E**). Apoptosis analysis showed that sh-SPARC cells exhibit higher apoptosis levels than the sh-NC group in **Figure 7C**. Furthermore, ablation of ITGB1 decreased cell viability significantly more than the NC group, and overexpression of ITGB1 increased cell viability more than in the NC group (**Figures 9A** **–C**).

In **Figure 9A**, the tube formation test has been investigated among the NC, sh-SPARC, sh-ITGB1, and sh-SPARC-ITGB1 groups. The corresponding image was shown, and the results revealed that the sh-SPARC, sh-ITGB1, and sh-SPARC-ITGB1 groups could reduce tube formation rate and tube area covered than the NC group (**Figures 9B, C**).

## Discussion

DR is the most common complication of diabetes mellitus, and it is a leading cause of vision impairment and blindness (24). The present study firstly shows that high expression of SPARC has been detected in serum and vitreous samples of DR patients. The second part showed that HG-induced HRCEC cells could upregulate the SPARC/ITGB1 pathway signaling and inflammation biomarkers when combined with the GEO dataset. Finally, ablation of SPARC and ITGB1 can also regulate apoptosis, cell viability, and tube formation.

C-reactive protein, *N*-epsilon-carboxymethyl lysine (*N*-ϵ-CML), and pentosidine are just a few of the many circulating, vitreous, and genetic biomarkers that have been studied lately (25). VEGFR is a currently recognized biomarker of DR and plays a key role in the pathogenesis of DR by stimulating angiogenesis (26). However, not all patients respond to anti-VEGF therapy, and the treatment has side effects, such as short duration of action and the repetitive need for intraocular injection (27). Therefore, the identification of a new biomarker for DR is necessary to increase detection, risk stratification, and treatment for patients with DR.

SPARC plays a crucial role in the development of many diseases, including cancer and cardiovascular, osteoarticular, and metabolic diseases. Li et al. found that SPARC and FN1 are highly expressed and significantly related to the poor prognosis of gastric adenocarcinoma (28). Furthermore, SPARC is a key mediator of TGF-β-induced renal cancer and metastasis (29). In NAFLD-associated hepatocellular carcinoma, the inhibition of SPARC accelerates the development of cancer and cardiovascular disease (30). SPARC contributes to myocardial fibrosis in pressure overload (31). SPARC also plays a potential role in load-induced regulation of tendon homeostasis, vertebrate cartilage mineralization, and bone healing (32–34). SPARC is required to maintain glucose homeostasis and insulin secretion in mice with metabolic diseases such as obesity and type 2 diabetes (9). Previous studies found that SPARC plays an important role in hyalocyte-to-myofibroblast transdifferentiation in proliferative diabetic retinopathy (10). However, more in-depth studies on the role of SPARC in diabetic retinopathy are currently lacking.

In our studies, we found that ablation and overexpression of SPARC regulate the expression of ITGB1, inflammation, and VEGFR levels, which contribute to changes in apoptosis, cell viability, and tube formation. As mentioned in the literature review, SPARC influences skeletal muscle-derived satellite cell migration and differentiation through the ITGB1-mediated signaling pathway (20). ITGB1 and SPARC exhibit lens epithelial cell-like characteristics in cataracts (21). These results corroborate the findings of a lot of our studies that SPARC may play an important role in regulating the ITGB1-mediated signaling pathway. SPARC also regulated the expression of VEGFR in DR. Previous studies have shown that SPARC regulates glioblastoma growth by altering the tumor microenvironment and inhibits tumor angiogenesis by inhibiting VEGF expression and secretion (15). Sachiko et al. investigated that SPARC activates fibroblasts only in the presence of fibronectin, which is abundantly secreted in endometrial cancer cells expressing SPARC, and SPARC-FN1-mediated fibroblast activation may be associated with increased cancer cell migration and invasiveness (35). In addition, fibronectin acts as a molecular switch that determines SPARC function in pancreatic cancer (36). SPARC also regulates the inflammation response *via* targeting TGF-β, which is also consistent with our results (37).

SPARC protein could also regulate cell apoptosis, cell viability, and tube formation. SPARC deficiency suppresses diabetes-stimulated increases in superoxide production and eliminates prominent features of hepatocyte damage, such as impaired cytoprotection, inflammation, apoptosis, and autophagy (38). SPARC increases NOX4 expression *via* a TGF-β1-dependent signaling pathway, leading to oxidative stress and proinflammatory matrix behavior and apoptosis in human brain smooth muscle cells (39). The downregulation of SPARC could decrease cell migration, invasion, and viability (40). SPARCs are targeted antiangiogenic proteins in dysfunctional endothelial colony-forming cells that have distinct proteomic profiles and phenotypic changes when compared with hyperangiogenic endothelial cells with impaired angiogenesis and dilation (41). SPARC promotes apoptosis of neurovascular cells, including astrocytes, and is implicated in the pathogenesis of neurovascular rupture-focused diseases (41). Furthermore, SPARC was confirmed to be extremely important in promoting vascular endothelial cell proliferation, motility, and capillary−like tube formation, as well as reducing apoptosis (42). Therefore, this finding broadly supports the findings of other studies in this field, which have linked SPARC with cell apoptosis, cell viability, and tube formation in DR.

In HG-induced retinal pigment epithelium in a dose-dependent manner, HG promoted reactive oxygen species (ROS) production and apoptosis and inhibited autophagy and proliferation, while low glucose induced ROS production and autophagy but had little effect on apoptosis and proliferation (43). Consistent with the literature, this research found that participants who reported using HG-induced retinal pigment epithelium played an important role in the development of DR. In addition, previous studies showed that HG-induced HRCEC inhibition of cell viability, migration, angiogenesis, and cell adhesion was reversed by inhibition of SPARC expression (44). In our studies, we found that HG-induced apoptosis can be reversed by ablation of SPARC.

We also use bioinformatic tools to analyze the key genes in DR as we previously described (45). These results revealed that SPARC is markedly associated with STAB1, VEGFR, ILK, and FN1. In-depth transcriptomic analysis of the human retina reveals molecular mechanisms such as SPARC underlying diabetic retinopathy (46). It is important to consider the possibility of bias in these responses from verified bioinformatic analysis.

## Conclusion

Overall, our study indicates that SPARC mediates the expression of ITGB1, which plays an important role in regulating cell apoptosis, cell viability, and tube formation *in vitro* experiments. The results of this study will help in understanding the pathogenesis of DR, the development of effective drugs, and the development of a more comprehensive and effective strategy for the prevention and treatment of DR.

## Data Availability Statement

The datasets presented in this study can be found in online repositories. The names of the repository/repositories and accession number(s) can be found in the article/**Supplementary Material**.

## Ethics Statement

The studies involving human participants were reviewed and approved by the ethics committee of Shanghai General Hospital. The patients/participants provided their written informed consent to participate in this study.

## Author Contributions

Conception and design: ZZ, YF, MT, and YYJ. Acquisition, analysis, and interpretation of the data: LYL, XS, JHW, SMC and TWQ. Drafting and writing: ZYG. Final approval of the article: LYL. All authors listed have made a substantial, direct, and intellectual contribution to the work and approved it for publication.

## funding

This project was qualified by the Reaserch Fund of Shanghai Tongren Hospital, Shanghai Jiaotong University School of Medicine (NO:TRYJ2021JC02).

## Conflict of Interest

The authors declare that the research was conducted in the absence of any commercial or financial relationships that could be construed as a potential conflict of interest.

## Publisher’s Note

All claims expressed in this article are solely those of the authors and do not necessarily represent those of their affiliated organizations, or those of the publisher, the editors and the reviewers. Any product that may be evaluated in this article, or claim that may be made by its manufacturer, is not guaranteed or endorsed by the publisher.

## Supplementary Material

Supplementary Figure 1 GO pathway signaling analysis in GEO dataset. **(A)** GO network analysis. **(B)** GO diseased analysis. **(C)** Target genes of GO anlayais. **(D)** The regulated genes analyzed by GO dataset.
