# Supplementary figures and images for: Secreted Protein Acidic and Rich in Cysteine Mediates the Development and Progression of Diabetic Retinopathy
Source: Front Endocrinol (Lausanne). 2022 Jun 3;13:869519. doi: 10.3389/fendo.2022.869519 (PMC9205223; doi:10.3389/fendo.2022.869519)

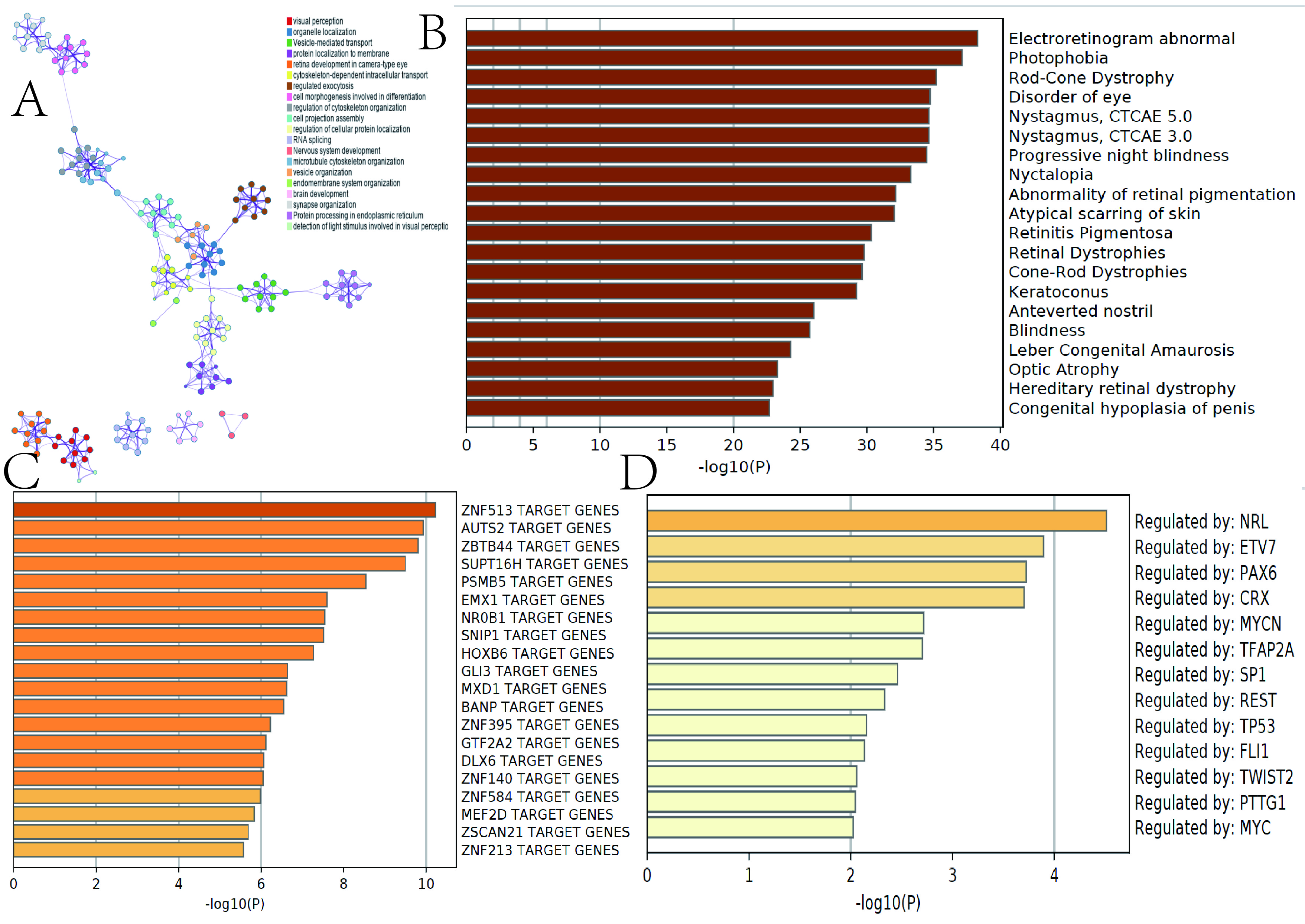

Supplement: Supplementary Figure 1 — GO pathway signaling analysis in GEO dataset. (A) GO network analysis. (B) GO diseased analysis. (C) Target genes of GO anlayais. (D) The regulated genes analyzed by GO dataset. [file Image_1.jpeg]
